# Influence of Surface and Bulk Defects on Contactless Resistivity Measurements of CdTe and Related Compounds

**DOI:** 10.3390/s20154347

**Published:** 2020-08-04

**Authors:** Jan Franc, Roman Grill, Jakub Zázvorka

**Affiliations:** Faculty of Mathematics and Physics, Institute of Physics, Charles University, Ke Karlovu 5, CZ-121 16 Prague 2, Czech Republic; grill@karlov.mff.cuni.cz (R.G.); zazvorka@karlov.mff.cuni.cz (J.Z.)

**Keywords:** contactless resistivity, CdTe, surface defects, nonexponential response

## Abstract

We analyzed the influence of parameters of deep levels in the bulk and conditions on the surface on transient charge responses of semi-insulating samples (CdTe and GaAs). We studied the dependence on the applied bias step used for the experimental evaluation of resistivity in contactless measurement setups. We used simulations based on simultaneous solutions of 1D drift diffusion and Poisson’s equations as the main investigation tool. We found out that the resistivity can be reliably determined by the transient contactless method in materials with a large density of deep levels in the bulk (e.g., semi-insulating GaAs) when the response curve is described by a single exponential. In contrast, the materials with the low deep-level density, like semiconductor radiation detector materials (e.g., CdTe, CdZnTe, etc.), usually exhibit a complex response to applied bias, depending on the surface conditions. We show that a single exponential fit does not represent the true relaxation time and resistivity, in this case. A two-exponential fit can be used for a rough estimate of bulk material resistivity only in a limit of low-applied bias, when the response curve approaches a single-exponential shape. A decreasing of the bias leads to a substantially improved agreement between the evaluated and true relaxation time, which is also consistent with the approaching of the relaxation curve to the single-exponential shape.

## 1. Introduction

Contactless methods of resistivity measurement are widely applied in development of materials used in the industry—GaAs, GaN, SiC, CdTe and related compounds and others. A frequently used method for semi-insulating semiconductors is a capacitive technique that relies on transient charge measurements in the time domain of a sample positioned in an air capacitor. It was developed and tested on semi-insulating GaAs wafers [1] and was originally referred to as “time-dependent charge measurement—TDCM”. Later, an acronym, Corema (Contactless Resistivity Mapping), became more common. Due to its simplicity and possibility to measure large wafers in a mapping mode, it has been widely applied on different materials—InP [2,3], SiC [4] and mainly CdTe and related compounds—e.g., [5,6]. The evaluation of resistivity from the measured transient response is based on a model assuming that the response is single exponential. In this case, the analysis of resistivity is simple, and when combined with the simplicity of the measurements, the method represents a powerful tool for fast characterization of resistivity distribution in large wafers. However, in many cases, an experiment nonexponential transient response is observed, and a more complex approach is necessary to analyze the data. The goal of this paper is to check theoretically and experimentally the applicability of the technique from the point of view of prerequisites demanded by the theory and the evaluation of a response produced by a real setup. We use simulations based on a simultaneous solution of 1D drift diffusion and Poisson’s equations as the main tool and demonstrate the conclusions of simulations with some experimental measurements.

## 2. Materials and Methods

**Figure 1:**
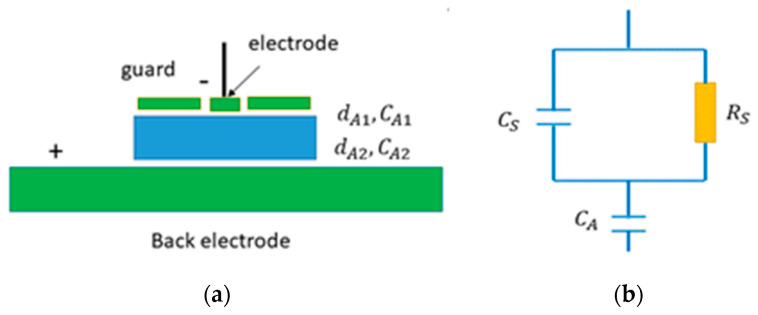
Basic scheme of the Contactless Resistivity Mapping (Corema) setup (**a**) and the equivalent circuit (**b**) *C_A_* represents the total capacity of both air gaps.

According to the principal model of the contactless resistivity measurements [1], the sample characterized by resistance *R_S_* and capacity *C_S_* is placed between two electrodes, one laying on the bottom, while having an air gap between the top electrode and the sample. After application of the bias *U*, the electrodes act as a capacitor, and the whole system consisting of the material and the two air gaps with thicknesses $d_{A1}\;\mathrm{and}\;d_{A2}$ is charged (Figure 1a). The bottom air gap $d_{A2}$ and serial capacity $C_{A2}$ are canceled when the sample rests directly on the back electrode. Therefore, the air gap width is $d_{A}\approx d_{A1}$ and its capacity $C_{A}\approx C_{A1}.$ The free carriers drift to the surfaces of the sample after bias application, where they electrically compensate the charge at the air gaps. The whole process in an ideal case has a single exponential character described by a time constant *τ_0_* [1]. The resistivity is then calculated by formula

(1) $$\rho =\frac{Q_{0}\tau_{0}}{\varepsilon_{0}\varepsilon_{r}Q_{\infty }}$$

where $Q_{0}$ is the initial charge induced at the onset of the bias on the serial capacity of the air gaps and the material. $Q_{\infty }$ is the terminal charge on the capacitor consisting of the capacity of the air gaps, *τ* is the charge relaxation parameter and $\varepsilon_{0}$ and $\varepsilon_{r}$ are the vacuum permittivity and material relative permittivity, respectively. $\varepsilon_{r}=10.3$ used for CdTe here.

The applicability of the above-mentioned theory and the correct evaluation of the resistivity by Equation (1) is conditioned by the fulfillment of three principal prerequisites:

1. The transient charge must be supplied at the sample’s surface in a sufficient amount so that no carrier depletion appears after the biasing.
2. The sample’s interface must act as an ideal ohmic-type contact at the charge supply so that neither carrier injection nor depletion appears.
3. The surface conductivity must be low so that the surface shunt may be effectively suppressed by the guard.

We focus now on the items 1 and 2 in detail.

1. The equivalent circuit of the setup shown in Figure 1b, allowing the derivation of Equation (1), depicts the investigated material by a couple of ideal devices—resistor *R_S_* and capacitor *C_S_*. Once the circuit has to fit a real process in the system after biasing, the charge must be supplied either by the contact with the back electrode or by the sample surface at the gap. Typically, one of the interfaces, further denoted as a leading interface, plays a more important role at the charge supply. The assignation of the leading interface depends on the type of conductivity of the measured material and the polarity of the bias—cathode (anode) in the n-type (p-type). A strong generation and recombination of the electron-hole pairs in the leading interface should preserve the carriers’ source in the thermodynamic equilibrium, regardless the drain of the charge after biasing. When the high resistivity material with a mixed conductivity is measured, both interfaces play a role of the leading interface at the charge supply.

The dynamics of free carrier generation at the surface may be conveniently characterized by the surface recombination velocity *s* reflecting the surface source through the balanced equation

(2) $$E\left(0\right)\mu \left(n_{\;}-∆n\right)=s∆n$$

where *E*(0) is the electric field at the surface after the switch-on of the bias, *µ* is the carrier mobility, *n* is the equilibrium density of the majority carriers in an unbiased sample, and ∆*n* is the drop of the carrier density at the surface caused by the biasing. The left-hand side of Equation (2) depicts the drain of free carriers from the sample’s surface due to the bias; the right-hand side defines the source of carriers generated at the surface. In an ideal case complying to the circuit in Figure 1b, the surface carrier density must not significantly deviate from *n*, so that ∆n≪ *n*. The requirement for *s* is then defined by the clause

(3) $$s\gg \frac{C_{A}}{C_{A}+C_{S}}\frac{\mu U}{L}$$

where E(0) was expressed in agreement with the circuit in Figure 1b as $E\left(0\right)=\frac{C_{A}}{C_{A}+C_{S}}\frac{U}{L}$, where U and *L* are the bias applied between the electrodes and wafer thickness, respectively. Considering typical values of $d_{A}\sim 200$ μm, L~2 mm and U≈5V and characteristic values of $\frac{C_{A}}{C_{A}+C_{S}}\approx 0.5$ and electron mobility in CdTe $\mu_{e}\approx 1000\;{\mathrm{cm}}^{2}/\mathrm{Vs}$, we obtain the condition for surface recombination velocity $s\gg 2.5\times 10^{4}\;\mathrm{cm}/s$. For the case of a surface recombination of holes with mobility $\mu_{h}\approx 80\;{\mathrm{cm}}^{2}/\mathrm{Vs},$ we obtain the condition $s\gg 10^{3}\;\mathrm{cm}/s$.

Measurements of the surface recombination velocity in detector-grade CdTe are scarce. Another important application of CdTe are solar cells [7,8,9], where most of the work of research of surface recombination velocity was done. The recombination velocity $s\approx 1.6\times 10^{5}\;\mathrm{cm}/s$ determined in untreated crystal was reported [10], while it decreased to $s\approx 1.4\times 10^{4}\;\mathrm{cm}/s$ after surface treatment. Rather low $s\approx 1.1\times 10^{4}\;\mathrm{cm}/s$ may be deduced from the measurements of photovoltage spectra [11]. According to [12], *s* is of the order of magnitude of 10^2^ cm/s in semiconductors with an etched surface. The complexity of the surface recombination in silicon was illustrated in a previous study [13]. We conclude that, in most cases of surface treatment and under standard measuring conditions (applied bias *U* and sample thickness *L*), there is a high risk that Equation (3) does not hold. In this case, Equation (1) cannot be applied to evaluate resistivity.

2. Procedures routinely applied at the preparation of samples for contactless resistivity measurements involve versatile technological steps affecting to some degree the surface of the wafer (cutting, grinding, polishing, etching and passivation). The creation of multiple electrically active defects localized near the surface may be expected. The appearance of additional uncompensated charge defects yields a deviation of the Fermi energy from the middle of the band gap, band bending and space charge formation near the material surface. In this case, the bias of a few volts, which is typically applied in the measurement setup, tends to be large enough to work up the deviation from the ideal ohmic character of the surface charge source and leads to an appearance of nonlinearities in the charging process.

Concluding from this analysis, we emphasize the fact that the source of carriers at the leading interface, supplying free carriers into the biased bulk, may deviate from the ideal ohmic-type contact characteristics. Both an excessive and a deficient supply may be expected. While the former eventuality leads to a systematically lowered relaxation time *τ* and, therefore, resistivity *ρ*, the latter option results in an enhanced *τ* and *ρ*.

An alternative way to create the available charge could appear in a material possessing a deep level with a high density and capture cross-section positioned near the Fermi level in the sample’s bulk, i.e., without the particular surface source. We analyze this eventuality more deeply at the end of Section 3.2, where, where we compare the charge dynamics in the detector-grade CdTe and in a material possessing bad detection performance.

In the next section, we present that violations of the Corema prerequisites result in visible deviations from the linearity demonstrated by a non-single-exponential relaxation. Generally, we may observe two regimes characterizing the relaxation: (i) an accelerated relaxation appearing in the case of carrier injection. This process may be conveniently fitted with the sum of two damped exponentials according the formula

(4) $$Q\left(t\right)=Q_{0}+\left(Q_{\infty }-Q_{0}\right)\left(1-\sum_{i=1}^{2}a_{i}e^{-\frac{t}{\tau_{i}}}\right)$$

where the sum of amplitudes satisfies $a_{1}+a_{2}=1$. Respective relaxation terms τ*_i_* depict the initial fast relaxation and the follow-up retardation of the process. (ii) A slowed-down relaxation induced by an insufficient source of free carriers. This state occurs either due to an insufficient carrier generation at the surface with the low surface recombination, see Equation (3), or at the carrier depletion incurred by the blocking leading interface. The relaxation in such a process cannot be fit with Equation (4), since the slow component of the relaxation dominates at the initial period of the process. It has appeared that a convenient trial function proper for the fit of such processes may be defined by

(5) $$Q\left(t\right)=Q_{0}+\left(Q_{\infty }-Q_{0}\right)\left(1-\frac{1}{\sum_{i=1}^{2}a_{i}e^{\frac{t}{\tau_{i}}}}\right)$$

where all parameters retain the features analogous to those in Equation (4).

Appropriately chosen trial functions defined by Equations (4) and (5) allowed us excellent fits of all curves calculated at the numerical simulations. The deviation of the fit from the fitted curves was less than 1% in all cases. The coefficient of determination of the fit was $R^{2}>0.99999$ in all cases.

## 3. Results

### 3.1. Numerical Simulations

In this section, we analyze several examples characterizing different surface conditions of samples described by the efficiency of the surface source of free carriers and/or by band bending. We perform numerical simulations of the charge dynamics solving one-dimensional drift-diffusion and Poisson’s equations. Both electrons and holes are involved. The free carrier interaction with impurity levels is described by the Shockley-Read-Hall model [14]. Details of the approach may be found in [15]. Since the relaxation time *τ* is sufficiently fast here, the full time-integration involving the electron and hole transients is used. The nonsymmetrical boundary conditions simulating the contactless setup are distinguished by an ideal metal contact on one side representing the back electrode and a free surface as the air gap on the other side. The metal contact is characterized by the work function of the metal defining the band bending. Free carriers are permanently in the thermodynamic equilibrium at the semiconductor-metal (MS) interface in the drift-diffusion model. An eventual appearance of an insulating layer forming the metal-insulator-semiconductor (MIS) structure is omitted. The air gap is defined by a prescribed zero current across the interface.

Two types of defect levels are considered. Level A defines defects homogeneously spread through the whole volume of the sample. Level B is confined to a narrow surface layer below the air gap. The generation of free carriers at the free surface may be mediated in this way. Each defect level is characterized by its position in the band gap, defect density and electron and hole capture cross-sections.

With the aim to illustrate the peculiarities of transient effects at the contactless setup, we performed all simulations on a p-type CdTe at temperature 295 K characterized with fixed transport parameters and the defect structure of the bulk. The chosen parameters represent a typical high-resistivity single-crystalline detector-grade sample, with the Fermi level close to the mid-gap, experimentally observed mobilities of electrons and holes [16] and a deep level fixing the Fermi level while guaranteeing sufficiently a long lifetime of the free carriers. The parameters used are as follows: energy gap $E_{g}=1.51\;\mathrm{eV}$, Fermi energy $E_{F}=E_{C}-0.785\;\mathrm{eV}=-0.52E_{g}$, equilibrium electron and hole density $n=2.8\times 10^{4}\;{\mathrm{cm}}^{-3}$ and $p=8\times 10^{6}\;{\mathrm{cm}}^{-3}$ and electron and hole mobility $\mu_{e}=10^{3}{\;\mathrm{cm}}^{2}/\mathrm{Vs}\;\mathrm{and}$ $\mu_{h}=77\;{\mathrm{cm}}^{2}/\mathrm{Vs}$. We use a single deep level defined by the density $N_{t}=10^{11}\;{\mathrm{cm}}^{-3}$, energy $E_{t}=E_{F}$ and equal capture cross-section of electrons and holes $\sigma_{e}=\sigma_{h}=10^{-15}{\;\mathrm{cm}}^{2}$. The resistivity results $\rho =9.7\times 10^{9}\;\Omega \mathrm{cm}$. The degeneracy factors of all considered defect levels are equal to one for simplicity. The thickness of the simulated sample *L* = 2 mm and the thickness of the air gap $d_{A}=0.2\;\mathrm{mm}$ are chosen, which define the ratios of respective capacities and charges as

(6) $$\frac{Q_{0}}{Q_{\infty }}=\frac{C_{S}}{C_{A}+C_{S}}=0.507$$

The relaxation time corresponding to chosen *ρ* and $\frac{Q_{0}}{Q_{\infty }}$ is $\tau_{0}=17.44\;\mathrm{ms}$. Surfaces of semiconductor samples typically contain higher concentrations of defects and connected energy levels in the bandgap. Therefore, even in a detector-grade sample with a low concentration of deep mid-gap levels ($N_{deep}\sim 10^{11}{\;\mathrm{cm}}^{-3})$ in the bulk, the surface concentration of these levels can be much higher ($N_{deep}^{surf}\sim 10^{16}{\;\mathrm{cm}}^{-3})$. We included such a thin layer in some examples and discuss its effects to the charge relaxation and evaluated mobility.

**Figure 2:**
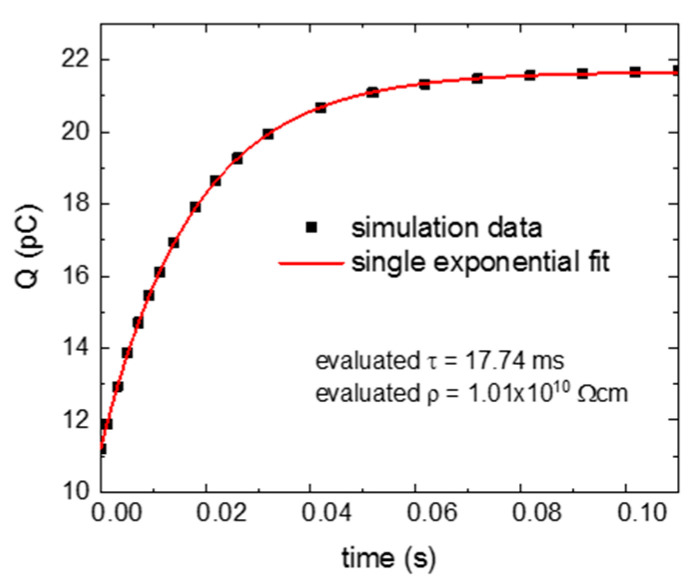
Numerical simulation and fit of a collected charge *Q* on a sample with flat bands and with an ideal supply of majority free holes from the leading surface to the bulk. The fit yields resistivity $\rho =1.01\times 10^{10}\;\Omega \;\mathrm{cm}$, which is nearly the same as the input resistivity calculated from the sample parameters—$\rho =9.7\times 10^{9}\;\Omega \;\mathrm{cm}$. Simulation and fit curves are practically the same, in this case. The simulation curve is represented only by chosen points to distinguish it from the fit. The coefficient of determination of the fit is $R^{2}=0.99999$.

We start the simulations with an ideal case, when the leading interface with flat bands forms an ohmic contact with the back electrode, i.e., it provides a sufficient supply of free holes to the sample, keeping their equilibrium concentration during the whole time of the sample response to the applied voltage step. The results of the simulation and its single exponential fit are presented in Figure 2. It is evident that the response can be well-described by a single exponential fit, and the evaluated resistivity is practically the same as the input one. The slight increase of the resistivity is caused by a depletion of minority electrons, which are not formed in sufficient amounts at the ideal defect-free surface (cathode) adjacent to the air gap.

**Figure 3:**
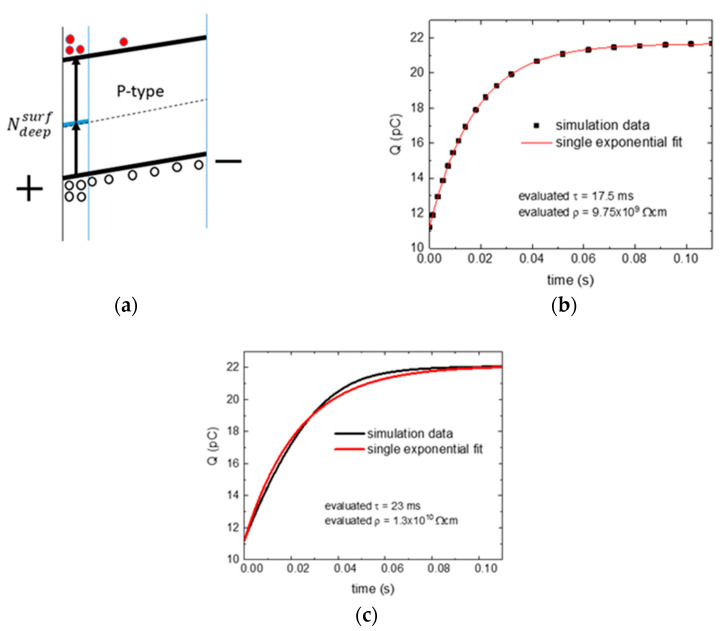
Surface source with flat bands (**a**) basic scheme and (**b**) time evolution of the collected electric charge with a surface source (deep level of type B)—$N_{deep}^{surf}=2\times 10^{16}\;{\mathrm{cm}}^{-3}$ and $\sigma_{e}=\sigma_{h}=10^{-15}\;{\mathrm{cm}}^{2}$. Simulation and fit curves are practically the same, in this case. The simulation curve is represented only by chosen points to distinguish it from the fit. The coefficient of determination of the fit $R^{2}=0.99999$. (**c**) The time evolution of the collected electric charge with a surface source $N_{deep}^{surf}=2\times 10^{14}\;{\mathrm{cm}}^{-3}\;\mathrm{and}\;\sigma_{e}=\sigma_{h}=10^{-15}\;{\mathrm{cm}}^{2}$. The coefficient of determination of the fit $R^{2}=0.99816$.

The following two simulations describe the impact of the surface source of free carriers on the response of the sample to a voltage step of 5 V and on the evaluation of relaxation time *τ* and resistivity *ρ*. Oppositely to other examples shown in this section, the anode is set to the free surface, which thus becomes the leading interface here. Figure 3a shows the basic scheme of a sample with a surface source of free carriers and with flat energy bands. Figure 3b,c presents the results of the numerical simulation of the relaxation response for two surface sources with concentrations of level B—$N_{deep}^{surf}=2\times 10^{16}{\;\mathrm{cm}}^{-3}$ (source I) and $N_{deep}^{surf}=2\times 10^{14}{\;\mathrm{cm}}^{-3}$ (source II) positioned at the same energy as the bulk defects distributed in a 10-µm thin layer near the surface. The capture cross-sections for electrons $\sigma_{e}=10^{-15}{\;\mathrm{cm}}^{2}$ and holes $\sigma_{h}=10^{-15}{\;\mathrm{cm}}^{2}$ equal to those in the bulk level are used. The level is half-by-half filled by electrons and defined initially neutral so that no space charge affects the free carrier supply. Nearly the same results were obtained with a thinner layer when the defect density was increased proportionally.

The time evolution of the collected charge after the application of the bias with source I can be well-fitted by a single exponential. The evaluated relaxation time τ=17.5 ms is nearly the same as the true sample input time $\tau_{0}=17.44\;\mathrm{ms}$. This situation corresponds to the case when the surface generation source suffices to supply carriers (in our model case, majority holes) that are extracted from the surface by the applied bias. The evaluated resistivity is correct.

Figure 3c shows the evolution of the collected electric charge for the weaker source II. The dependence is a complex function. The reason for this behavior can be described as follows. The source of the holes is insufficient to resupply all holes drained from the sample volume after application of the bias. The reduced hole density slows down the relaxation. Simultaneously, a negative space charge is formed due to the hole extraction. An excellent fit was obtained using Equation (5) with parameters $\tau_{1}=41.5\;\mathrm{ms}$, $a_{1}=0.805$, $\tau_{2}=12.7\;\mathrm{ms}$ and $a_{2}=0.195$, producing a curve practically undistinguishable from the fitted one. The considerable deviation of both $\tau_{i}$ (*i* = 1 and 2) from the correct $\tau_{0}$ is eminent.

**Figure 4:**
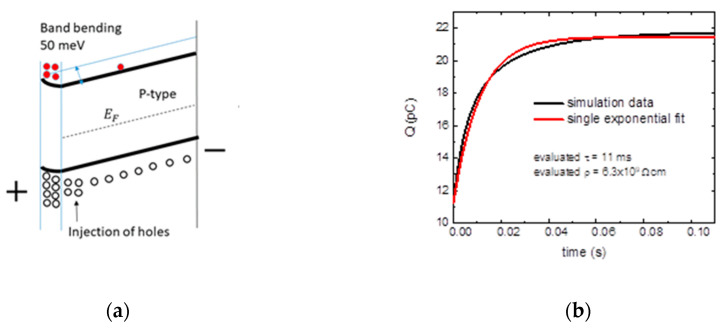
Surface source with band bending injecting holes at the anode (**a**) basic band scheme and (**b**) time evolution of the collected electric charge. $E_{F}$ represents the Fermi energy. The coefficient of determination of the fit is $R^{2}=0.99456$.

**Figure 5:**
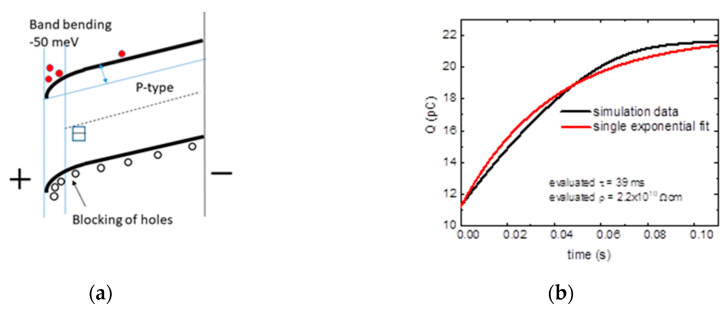
Surface source with band bending blocking holes at the anode (**a**) basic band scheme and (**b**) time evolution of the collected electric charge. The coefficient of determination of the fit is $R^{2}=0.99662$.

Let us now discuss the situation when the efficiency of the surface source is modified by the band bending. Next, the examples show the case of a p-type semiconductor with (i) bands bent up at the anode adjacent to the base electrode, injecting holes into the volume of the sample (Figure 4a), while (ii) bands bent down block the transport of holes to the volume (Figure 5a).

The surface source defined in example (I) above is used again to store holes in the thin surface layer after relaxation. This option is important when the simulations are performed at a low bias $U\approx k_{B}T/e$ and the Boltzmann-type free carrier distribution markedly spreads into the sample’s volume. The presence of the surface layer allows us to reach the final charge $Q_{\infty }$ defined by the setup geometry even at the low U.

If, in this situation, the bands are bent up, the back electrode is injecting holes in the volume. The simulated dependence of the collected charge on the time after application of the bias (Figure 4b) is characterized by a single exponential increase with a time constant smaller than the input *τ*. Therefore, the evaluated apparent resistivity is smaller than the real value.

If the bands are bent down, the surface is blocking the holes. Depending on the value of the band bending, a situation can occur when the surface source is no longer able to resupply the holes extracted after application of the bias inducing hole depletion. The transport and its characteristics have similar characters, as described in Figure 3c (flat bands and weak surface source of free carriers).

**Figure 6:**
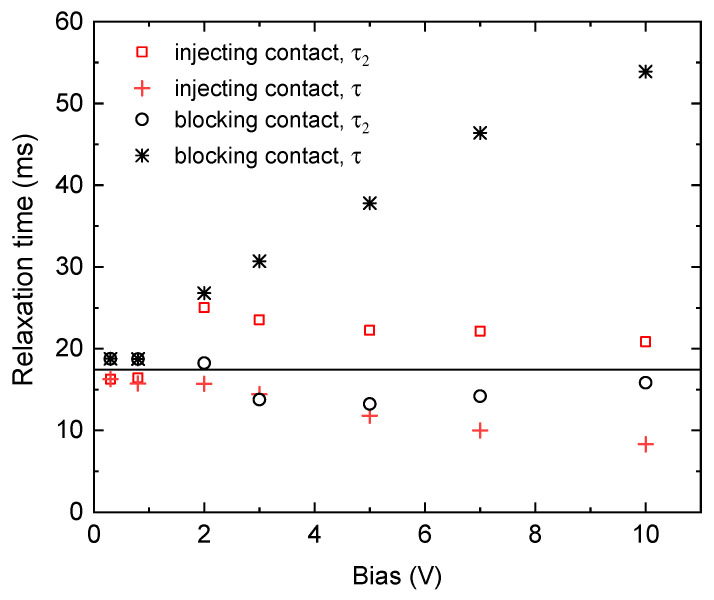
Dependence of the characteristic time of the charge relaxation derived by the fitting with Equations (4) or (5) (denoted as $\tau_{2}$) and the relaxation time τ obtained by the single-exponential fit of the charge relaxation on the applied bias. The input right relaxation time $\tau_{0}=17.44\;\mathrm{ms}$ is marked by the horizontal line.

It is apparent that single exponential fits do not provide sufficient agreement with the simulation data in many cases (Figure 3c, Figure 4b and Figure 5b), and the resistivity evaluated through Equation (1) markedly deviates from the correct value. Therefore, we applied trial functions (4) and (5), which involve two exponentials and compared the results of the fit with the true input relaxation time $\tau_{0}$. The goal was to assess whether this approach can lead to a better evaluation of the true relaxation time and this way to an estimate of the sample resistivity when its response to a bias step is nonexponential. We performed simulations in dependence of the applied bias in the range of 0.3–10 V for defect models with the hole injection and depletion schematized in Figure 4a and Figure 5a, respectively, and evaluated the results of the simulation using respective trial functions (4) and (5). We focused on the pertinent $\tau_{i}$ representing the relaxation at the later time where the disturbance caused by the band banding should fade away. That is, the longer (shorter) time denoted as $\tau_{2}$ in the case of injecting (blocking) an anode. The evaluated $\tau_{2}$ is presented in Figure 6. Simultaneously, we show the time *τ* received by the single-exponential fit.

We may see that *τ* deviates significantly from the right relaxation time $\tau_{0}$ represented by the horizontal black line in Figure 6 when the high bias is used. Decreasing of the bias leads to a substantially improved agreement between the evaluated and true relaxation times, which is also consistent with the approaching of the relaxation curve to the single-exponential shape. The evaluation of the relaxation by the double-exponential fits allowed us to approach to the $\tau_{0}$ even at the large bias. The deviation is mostly less than 30%. Nevertheless, as it is apparent at the injecting contact with U=2 V, anomalies caused by an interference between exponentials resulting in unwelcome instabilities may appear.

In the case where the leading interface is injecting holes (bands bent up 50 meV, Figure 4a), the value of $\tau_{2}$ is larger than $\tau_{0}$ at the higher applied bias, while, in the case of contact blocking, the majority holes (bands bent down 50 meV, Figure 5a) $\tau_{2}$ are shorter than *τ* at the higher applied bias. Using a low bias, $\tau_{2}$ approaches to the correct value and, at a very low bias, it even surpasses it. The deviation of *τ* from $\tau_{0}$ at the lowest bias is caused by the variation of the total equilibrium resistivity due to the band banding. In the case of blocking the anode, the depleted region contributes by a slightly damped relaxation. Conversely, the injecting contact yields an opposite effect, as is seen in Figure 6.

While the total relaxation velocity depicted by *τ* in the single-exponential fit accords with the prediction of the accelerated (decelerated) relaxation at the carriers’ injection (depletion), the $\tau_{2}$ evaluated with the two-exponential fit results oppositely, showing an extended (shortened) $\tau_{2}$ at these settings. This seeming disaccord comes from the interference of the two exponentials at the two-exponential fit. The exponential depicting the initial part of the relaxation through the relaxation time $\tau_{1}$ accommodates the principal period of the relaxation affected by the band bending and adopts partly also the latter stage of the relaxation at the quasi-ohmic regime at a low bias. Consequently, the second exponential is affected by this interplay, and the effect manifests in the observed behavior of $\tau_{2}$. This feature might be conveniently used at the rough simple estimation of the resistivity of the samples, considering that $\tau_{0}$ depicting the correct relaxation velocity should lie in the interval limited by $\tau_{2}$ and *τ*. While fitted $\tau_{2}$ may be used at the estimation of a correct $\tau_{0}$, the value of *τ* puts an additional clause on the error bar of $\tau_{0}$, as follows. If $\tau >\tau_{2}$, then $\tau >\tau_{0}>\tau_{2}$. If $\tau <\tau_{2}$, then $\tau <\tau_{0}<\tau_{2}$.

**Figure 7:**
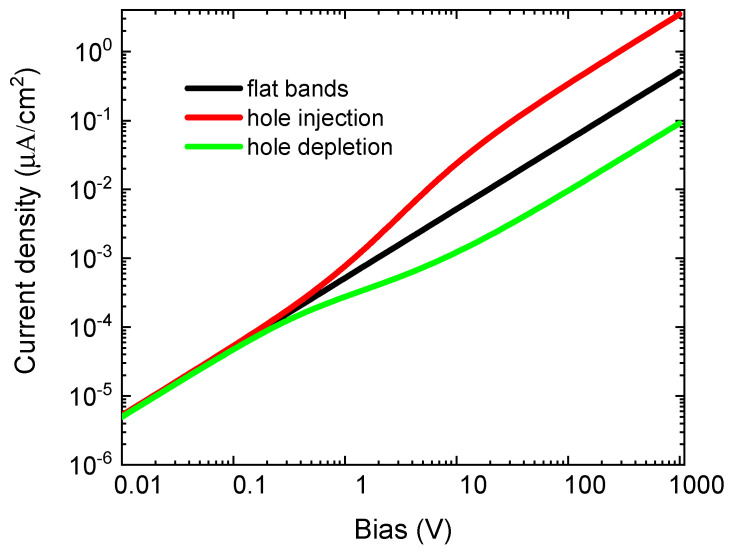
The I-V characteristics for flat bands at the leading surface (**black**), hole injection (bands bent up 50 meV, **red**) and hole blocking (bands bent down 50 meV, **green**). The mid-gap deep-level density was $N_{t}=10^{11}\;{\mathrm{cm}}^{-3}$.

The results shown in Figure 6 can be qualitatively understood from the simulated I-V characteristics for the studied example using flat bands, the injecting and the blocking regimes (Figure 7). Each point in the showed dependence was calculated by integrating the drift-diffusion equation 1 ms after the switch-on of the bias. The plotted current thus represents the initial current appearing at the contactless setup shortly after the biasing. We may see that the deviation from ideal ohmic-type I–V characteristics induced by band bending appears as early as at the biasing by 0.3 V. The deviation of the curves corresponding to the injecting and blocking regimes is substantial for the charge relaxation at the contactless setup. The non-ohmic character of the I–V characteristics results in the complex character of the charge relaxation.

We have used a rather small band bending of 50 meV at the leading interface in the simulations. If the band bending was larger, the observed effects of the carrier injection or depletion would be stronger. We also note that the reduction of the bias to reach the right *τ* may be partly avoided by measuring a thick wafer, at which the electric field related to the chosen bias is lowered. The maximum bias characterizing the ohmic part of the I–V characteristics is then increased.

**Figure 8:**
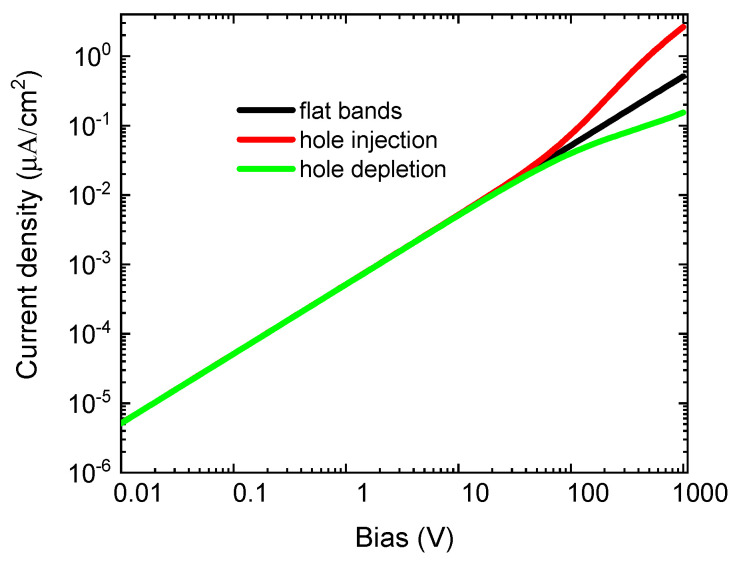
The I-V characteristics for flat bands at the leading surface (**black**), hole injection (bands bent up 50 meV, **red**) and hole blocking (bands bent down 50 meV, **green**). The mid-gap deep level density was $N_{t}=10^{14}{\;\mathrm{cm}}^{-3}$.

The principal contribution to the discussion to determine reliably the resistivity of high-resistive materials by the contactless method ensues from Figure 8. It shows the I–V characteristics of the material characterized by parameters equal to those used in previous simulations in Figure 7, with an exception of the deep level density, which was increased to $N_{t}=10^{14}{\;\mathrm{cm}}^{-3}$, i.e., 1000× compared to the previous case. We may see that, in this case, the nonlinearity induced by the band bending moved to a larger bias that is well-above the bias applied usually during measurements (typically, 5–10 V). Consequently, a single exponential relaxation producing the correct resistivity is obtained.

The reason for this effect is a much narrower width of the space charge region (depletion width) in the material, with the enhanced deep-level density and related suppressed contribution of the contact’s resistance [17] to the total resistance in the circuit predicted by the diffusion theory of metal-semiconductor contacts [18]. The high density of the level simultaneously with the chosen capture cross-section also accords with the model of the strong deep trap mentioned as an alternative charging channel in the theory section. The carriers’ injection or depletion is then accommodated by the trap, and the current attains the ohmic-like character for the sufficient time to reach the relaxation.

In detector-grade CdTe-based materials, the density of the mid-gap level is very low ($10^{9}–10^{12}{\;\mathrm{cm}}^{-3})$ [19]. Simultaneously, the captured cross-sections of the mid-gap levels are typically less than $10^{-13}\;{\mathrm{cm}}^{2}$ [20]. The requirement for long-carrier lifetimes is, according to the Shockley-Read-Hall model [14], in direct contradiction to the efficient thermal generation of electron-hole pairs via a deep level in the volume of the sample. Therefore, the alternative charging by Shockley-Read generation via deep levels does not take place.

### 3.2. Experimental

**Figure 9:**
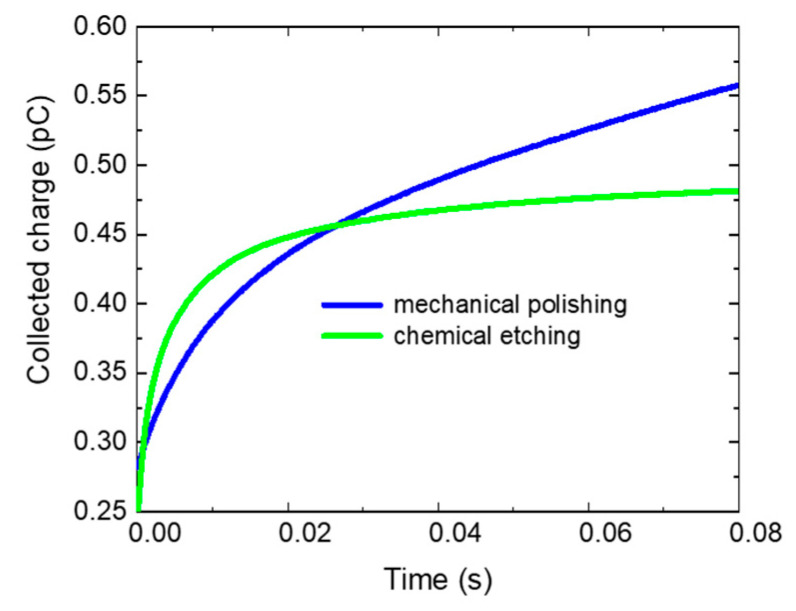
Experimental data for a CdZnTe sample with a surface treated by mechanical polishing and chemical etching.

To demonstrate that the modification of surface conditions can substantially influence the response of the sample to the applied bias, we measured a CdZnTe sample with two different surface treatments—mechanical polishing using a 1-μm alumina (Al_2_O_3_) abrasive and chemical etching by immersion into a 3% bromine–methanol (Br–methanol) solution for two min. Figure 9 shows the charging characteristics of the sample after both treatments. A considerable difference in both characteristics is apparent. This result confirms the models of influence of the surface conditions (parameters of surface deep levels and band bending) on the charge transport and shape of the charging characteristics discussed in the previous section.

## 4. Conclusions

We conclude that the resistivity may be reliably determined by the contactless method in materials with a large density of deep levels—typically, semi-insulating GaAs. In this case, the charge transient response to the applied bias is the single-exponential function. In contrast, the materials with a low deep-level density, like the state-of-the-art high-quality CdTe-based semiconductor radiation detector materials, exhibit frequently a nonexponential response, and the evaluation of resistivity by this technique proves to be more complicated. Even a weak deviation from the ideal conditions may result in a significant error of the evaluated resistivity. Based on the analysis done in this paper, we see two ways to solve the problem. One is to develop a surface treatment that minimizes the injection or depletion of carriers to the bulk or to decrease the applied bias to such a value that the response curve becomes a single exponential. Depending on the state of the surface and parameters of the defects in the bulk, this approach may require an application of biases below the currently used values in commercial setups.
